# Enhancing the Sensor Node Localization Algorithm Based on Improved DV-Hop and DE Algorithms in Wireless Sensor Networks

**DOI:** 10.3390/s20020343

**Published:** 2020-01-07

**Authors:** Dezhi Han, Yunping Yu, Kuan-Ching Li, Rodrigo Fernandes de Mello

**Affiliations:** 1 College of Information Engineering, Shanghai Maritime University, Shanghai 201306, China; dzhan@shmtu.edu.cn (D.H.); yu850139@163.com (Y.Y.); 2 Department of Computer Science and Information Engineering, Providence University, Taichung 43301, Taiwan; 3 Department of Computer Science, University of Sao Paulo, Sao Carlos, SP 13566-590, Brazil; mello@icmc.usp.br

**Keywords:** wireless sensor networks, DV-Hop, differential evolution, node localization

## Abstract

The Distance Vector-Hop (DV-Hop) algorithm is the most well-known range-free localization algorithm based on the distance vector routing protocol in wireless sensor networks; however, it is widely known that its localization accuracy is limited. In this paper, DEIDV-Hop is proposed, an enhanced wireless sensor node localization algorithm based on the differential evolution (DE) and improved DV-Hop algorithms, which improves the problem of potential error about average distance per hop. Introduced into the random individuals of mutation operation that increase the diversity of the population, random mutation is infused to enhance the search stagnation and premature convergence of the DE algorithm. On the basis of the generated individual, the social learning part of the Particle Swarm (PSO) algorithm is embedded into the crossover operation that accelerates the convergence speed as well as improves the optimization result of the algorithm. The improved DE algorithm is applied to obtain the global optimal solution corresponding to the estimated location of the unknown node. Among the four different network environments, the simulation results show that the proposed algorithm has smaller localization errors and more excellent stability than previous ones. Still, it is promising for application scenarios with higher localization accuracy and stability requirements.

## 1. Introduction

Wireless sensor networks (WSNs) are a form of network formed by freely organizing and combining tens of thousands of sensor nodes through wireless communication technology. It is a wireless network composed of several static or mobile sensors in a self-organizing and multi-hop way, aimed at exchanging the information processed by cooperative detection of perceived objects in the coverage area of the transmission network to users [1,2]. The wireless sensor has a far-reaching impact on human life and production as it integrates sensors, micro-electro-mechanical systems, and network communication technology [3,4,5]. At present, it has attracted significant attention from academia and industry and has become the focus and highlight of international competition. Localization technology is an essential supporting technology in wireless sensor networks where the estimated location information is crucial. A wide range of applications that require these easy-to-deploy, low-cost sensors well suited in WSNs includes military, agricultural, intelligent transportation, and environmental protection [6,7,8,9,10,11]. 

Although GPS can locate accurately, it is unrealistic to equip all micro sensor nodes with GPS in WSNs due to their high price and limited usage environment. Also, the localization accuracy of GPS in indoor and other complex environments may not be satisfactory [6]. Therefore, it is a challenging task to design an intelligent and effective localization algorithm under restricted conditions. Presently, research aims to take advantage of the interaction and connection between sensor nodes to achieve the goal of localization [12,13,14]. On the basis of the need to measure the distance between the actual nodes in the localization process, it can divide the localization algorithm into a range-based localization algorithm and a range-free localization algorithm [15,16]. The former needs to measure the absolute distance or azimuth between adjacent nodes and utilize the actual distance between nodes to calculate the localization of unknown nodes; such algorithms include time-of-arrival (TOA) [17], time-difference of arrival (TDOA) [18], angle of arrival (AOA) [19], and received signal strength indicator (RSSI) [20]. Regarding the latter, it is based on the network connectivity between nodes, utilizing the estimated distance between nodes to calculate the localization of nodes without measuring the actual distance between them; it includes algorithms Centroid [21], Distance Vector-Hop (DV-Hop) [22], Amorphous [23], Multi-Dimensional Scaling-programming (MDS-MAP) [24], and Approximate Point in Triangle Test (APIT) [25]. 

Range-free algorithms have the advantages of being low-cost, with low power consumption robust anti-measurement noise ability, and has simple hardware equipment; they can provide acceptable localization accuracy and, consequently, have attracted much attention in recent years [26]. DV-Hop, as one of the distributed localization methods based on distance vector routing and localization, is an algorithm that has attracted significant attention due to its simplicity and low equipment requirements [27].

There are two types of sensor nodes in WSNs: the anchor node with a known location, and the unknown node, the one to be located [28,29]. The purpose of the localization algorithm is to calculate the location of the unknown nodes in various ways. DV-Hop comprises three steps: the first step is that all nodes in the network get a hop-count value from the anchor nodes; next is calculated the average distance per hop for all anchor nodes; then, the estimated distance is obtained by multiplying the average distance per hop and the minimum hop-count value. Finally, the third step is to compute the localization of the unknown node according to the least-squares method [30,31], when the unknown node retrieves an estimated distance from three or more anchor nodes. The DV-Hop algorithm is easily implemented, and the localization accuracy it attains mainly depends on the estimated accuracy of average distance per hop and hop-count value between nodes. As known, the calculation error of the average distance per hop and the estimation error of hop-count value are the two primary sources of the estimation distance error.

On the basis of the abovementioned discussions, a wireless sensor node localization algorithm based on the improved DV-Hop and differential evolution (DE) algorithms is proposed in this paper, namely DEIDV-Hop. It can effectively reduce the localization error of nodes without increasing network traffic and hardware, and consists of three steps: the first two steps estimate the distance between unknown nodes and anchor nodes throughout information such as the average distance per hop and hop-count value, and the third step uses DE to determine the location of unknown nodes.

The main contributions of this paper include:

- The equation formula for calculating the average distance per hop of anchor nodes is improved. The minimum mean square error criterion more excellent to calculate the average distance per hop, and the average distance per hop of anchor nodes is obtained by applying its average value.
- DE algorithm has the defects of search stagnation and premature convergence; this improves the mutation formula and introduces random variation. In the crossover operation, the social learning part of the particle swarm optimization algorithm is introduced, and the heuristic information carried by the group optimal value is used to permit the current individual to learn from the group optimal individual, which is then intersected with the mutation vector. With the improvement of mutation operation and crossover operation of the basic DE algorithm, the diversity of the population is enhanced as well as information of the optimal individual in the population is effectively used to raise the efficiency of the algorithm.
- To validate and show the effectiveness of the proposed algorithm, the proposed DEIDV-Hop algorithm is evaluated under four different network environments. In comparison with known algorithms, DV-Hop, PSODV-Hop [32], and GSODV-Hop [33], experimental results show that the proposed DEIDV-Hop algorithm has smaller localization errors and greater stability, yet more suitable for application scenarios with higher localization accuracy and stability requirements.

The remainder of this paper is organized as follows. In Section 2, we summarize the related work, while in Section 3 we introduce the original DV-Hop and DE algorithms. The target algorithm DEIDV-Hop is presented in Section 4, then experimental results and performance evaluation are given in Section 5 and Section 6. Finally, the concluding remarks and future work direction of this paper are provided in Section 7.

## 2. Related Work

The range-free algorithm has several advantages: it is low cost and has low power consumption and simple hardware. DV-Hop localization algorithm has become an economical yet exceptionally important localization algorithm due to its simple algorithm, high coverage, and good feasibility that converts the estimated distance between nodes into the product of hop-count value and average distance per hop [32,33,34]. Even so, the disadvantage is that the localization accuracy is not good enough; accordingly, improvements in the localization accuracy are the key issues to this research. In this section, we introduce some related work of the DV-Hop localization algorithm.

Chen et al. proposed to estimate the distance of hop-count based on the value of neighbors in the same block [35]; to reduce the localization error, this study used weighted node distance to calculate the final location of nodes. Omar et al. used RSSI values to estimate the distance between the anchor node and the adjacent sensor node in one hop; such an estimated distance is applied to replace the average distance per hop in the original algorithm [31]. As a sensor node is located, it is elevated to act as an additional anchor node for subsequent use in other sensor nodes since the availability of the additional anchor node improves the accuracy of locating the remaining sensor nodes. Zhao et al. proposed a DV-Hop algorithm based on local weighted linear regression [35], where the unknown nodes attempt to estimate their locations by using nearby anchor nodes that maintain the proportional relationship between the geometric distance and the average distance per hop. The kernel method to improve the localization accuracy by increasing the weight of adjacent anchor nodes is applied.

Besides traditional optimization algorithms, many meta-heuristic algorithms to improve the localization algorithm have been considered, as they are competent to increase the accuracy of the original localization algorithm. Peng et al. proposed a genetic-based localization algorithm to improve the localization accuracy and convergence speed, limiting the feasible population region when the initial population was created. Three anchor nodes as the center to construct three squares are applied, in which the shadow area of the intersection of three squares is the overall workable area of the unknown nodes, and the initial population is generated randomly in the functional area. Singh et al. applied an improved algorithm for localization and then used particle swarm optimization to refine the results [36]. Zhao et al. proposed an improved localization algorithm based on hybrid chaotic strategy [10]. Harikrishnan et al. used the differential evolution algorithm to minimize localization error in wireless sensor network [12], while Cui et al. improved the values of hop-count by the values of common single-hop nodes between adjacent nodes and converted the discrete hop-count values into more accurate continuous values [37]. The differential evolution algorithm is introduced to achieve the optimal global solution corresponding to the estimated location of unknown nodes although it induces vast time overhead and energy consumption while improving the localization accuracy.

## 3. Distance Vector-Hop (DV-Hop) and Differential Evolution (DE) Algorithms

In this section, the process of the DV-Hop and DE algorithm is briefly introduced.

### 3.1. DV-Hop Algorithm

The three steps of the DV-Hop algorithm are discussed as follows.

Step 1: Flooding

In this step, all anchor nodes broadcast data packets with their localizations to their neighbors and hop digital segments are initially set to zero. The structure of the data packet is $\left\{id,x_{{}_{i}},y_{i},h_{i}\right\}$, including identifier id, the coordinate of the anchor node i is $\left(x_{i},y_{i}\right)$, $h_{i}$ the minimum hop-count value from the anchor node i, and the initial value of $h_{i}$ is zero. Once the neighbor node receives a packet with a smaller hop-count value from a particular anchor node, the localization of the anchor node is recorded and adds “1” to the hop-count value before sending it to other neighbor nodes [38,39,40,41]. Packets with high hop-count values are defined as obsolete information to be ignored. As soon as this step is completed, each one of the unknown nodes in the network retrieves the value of the minimum hop-count for each anchor node.

Step 2: Distance Estimation between Nodes

In this step, the distance between each node is estimated. First, it calculates the average distance per hop of each anchor node. For the anchor node, it uses Equation (1) to calculate the average distance per hop:

(1) $$HopSize_{i}=\frac{\sum_{i\neq j}d_{i,j}}{\sum_{i\neq j}h_{i,j}}$$

where $d_{i,j}=\sqrt{{\left(x_{i}-x_{j}\right)}^{2}+{\left(y_{i}-y_{j}\right)}^{2}}$, $\left(x_{i},y_{i}\right)$ and $\left(x_{j},y_{j}\right)$ represent the location of anchor nodes i and j, and $h_{i,j}$ represents the minimum hop-count value in anchor nodes i and j.

After calculating $HopSize_{i}$, each anchor node broadcasts its $HopSize_{i}$ in the system through controlled flooding. Equation (2) is used to determine the estimated distance between the unknown node and the anchor node i.

(2) $$d_{u,}{}_{i}=HopSize_{i}\times h_{u,i}$$

where $h_{u,i}$ represents the minimum value of hop-count between anchor node i and unknown node u.

Step 3: Estimate the Location of Nodes

In step 3, it is required to find out the location of all unknown nodes. For the unknown node u, the multilateration method [38] is used to estimate its location.

Let $\left(x_{u},y_{u}\right)$ be the location of the unknown node u and $\left(x_{i},y_{i}\right)$ be the location of the anchor node, where i=1,2,⋯,m. Therefore, the distance between unknown nodes u and m anchor nodes is specified by the following equation:

(3) $$\left\{ \begin{array}{l} {\left(x_{1}-x_{u}\right)}^{2}+{\left(y_{1}-y_{u}\right)}^{2}=d_{1}^{2}\\ {\left(x_{2}-x_{u}\right)}^{2}+{\left(y_{2}-y_{u}\right)}^{2}=d_{2}^{2}\\ \;\cdots \cdots \\ {\left(x_{m}-x_{u}\right)}^{2}+{\left(y_{m}-y_{u}\right)}^{2}=d_{m}^{2} \end{array}\right.$$

Equation (3) can be extended to:

(4) $$\left\{ \begin{array}{l} x_{1}^{2}-x_{m}^{2}-2\left(x_{1}-x_{m}\right)x_{u}+y_{1}^{2}-y_{m}^{2}-2\left(y_{1}-y_{m}\right)y_{u}=d_{1}^{2}-d_{m}^{2}\\ x_{2}^{2}-x_{m}^{2}-2\left(x_{2}-x_{m}\right)x_{u}+y_{2}^{2}-y_{m}^{2}-2\left(y_{2}-y_{m}\right)y_{u}=d_{2}^{2}-d_{m}^{2}\\ \cdots \cdots \\ x_{m-1}^{2}-x_{m}^{2}-2\left(x_{m-1}-x_{m}\right)x_{u}+y_{m-1}^{2}-y_{m}^{2}-2\left(y_{m-1}-y_{m}\right)y_{u}=d_{m-1}^{2}-d_{m}^{2} \end{array}\right.$$

The above Equation (4) can be expressed in matrix form as AX=B, where A, X and B are expressed by Equations (5)–(7), respectively.

(5) $$A=\left[\begin{array}{ll} 2\left(x_{1}-x_{m}\right) & 2\left(y_{1}-y_{m}\right)\\ 2\left(x_{2}-x_{m}\right) & 2\left(y_{2}-y_{m}\right)\\ \cdots \cdots & \\ 2\left(x_{m-1}-x_{m}\right) & 2\left(y_{m-1}-y_{m}\right)\; \end{array}\right]$$

(6) $$B=\left[\begin{array}{l} x_{1}^{2}-x_{m}^{2}+y_{1}^{2}-y_{m}^{2}+d_{m}^{2}-d_{1}^{2}\\ x_{2}^{2}-x_{m}^{2}+y_{2}^{2}-y_{m}^{2}+d_{m}^{2}-d_{2}^{2}\\ \cdots \cdots \\ x_{m-1}^{2}-x_{m}^{2}+y_{m-1}^{2}-y_{m}^{2}+d_{m}^{2}-d_{m-1}^{2} \end{array}\right]$$

(7) $$X=\left[\begin{array}{l} x_{u}\\ y_{u} \end{array}\right]$$

It utilizes the least square method to solve the equation. The coordinate location of the unknown node u is calculated:

(8) $$X={\left(A^{T}A\right)}^{-1}A^{T}B$$

### 3.2. Differential Evolution Algorithm

Differential evolution (DE) algorithm is a new evolutionary computing technology [13], designed to keep the population-based global search strategy of the evolutionary algorithm utilizing real coding, i.e., a simple mutation operation based on difference and one-to-one elimination mechanism to update the population that reduces the complexity of genetic algorithm operation.

#### 3.2.1. Initialization

DE uses NP D-dimensional real-valued parameter vectors as the population of each generation, and the t−th generation is denoted as $x_{i}^{t}=\left[x_{i,1}^{t},x_{i,2}^{t},\cdots ,x_{i,D}^{t}\right]$. Let the boundary of the parameter variable be $x_{j}^{\left(L\right)}<x_{j}<x_{j}^{\left(U\right)}$, the initial value of the i−th individual is generated by Equation (9).

(9) $$x_{i,j}^{0}=x_{j}^{\left(L\right)}+\left(x_{j}^{\left(U\right)}-x_{j}^{\left(L\right)}\right)*rand\left[0,1\right]$$

where rand[0,1] represents a uniform random number generated between [0,1], i=1,2,⋯,NP,j=1,2,⋯,D.

#### 3.2.2. Mutation

For each target individual $x_{i}^{t}$, where i=1, 2,⋯, NP, its mutant $v_{i}^{t+1}$ is generated by randomly select individuals that are used as the parent basis vector (rand) and a difference vector is used to generate the mutant individuals:

(10) $$v_{i}^{t+1}=x_{r_{1}}^{t}+F\left(x_{r_{2}}^{t}-x_{r_{3}}^{t}\right)$$

Among them, $r_{1}$, $r_{2}$ and $r_{3}$ represent random numbers and different unequal natural numbers in the range [1, NP]. F∈[0,2], as F represents the scaling factor.

#### 3.2.3. Crossover

The target individual $x_{i}^{t}$ and variant individuals $v_{i}^{t+1}$ are crossed to generate an experimental individual $u_{i}^{t+1}=\left[u_{i,1}^{t+1},u_{i,2}^{t+1},\cdots ,u_{i,D}^{t+1}\right]$. The crossover process is shown in Equation (11).

(11) $$u_{i,j}^{t+1}=\left\{ \begin{array}{l} v_{i,j}^{t+1},\;if\;r_{j}\leq CR\;or\;j=rnbr\_i\\ x_{i,j}^{t},\;otherwise \end{array}\right.$$

where $r_{j}$ is the j−th random number between [0,1]; rnbr_i is the random natural number between [1,D]; CR is the crossover rate, CR∈[0,1].

#### 3.2.4. Selection

**Figure 1:**
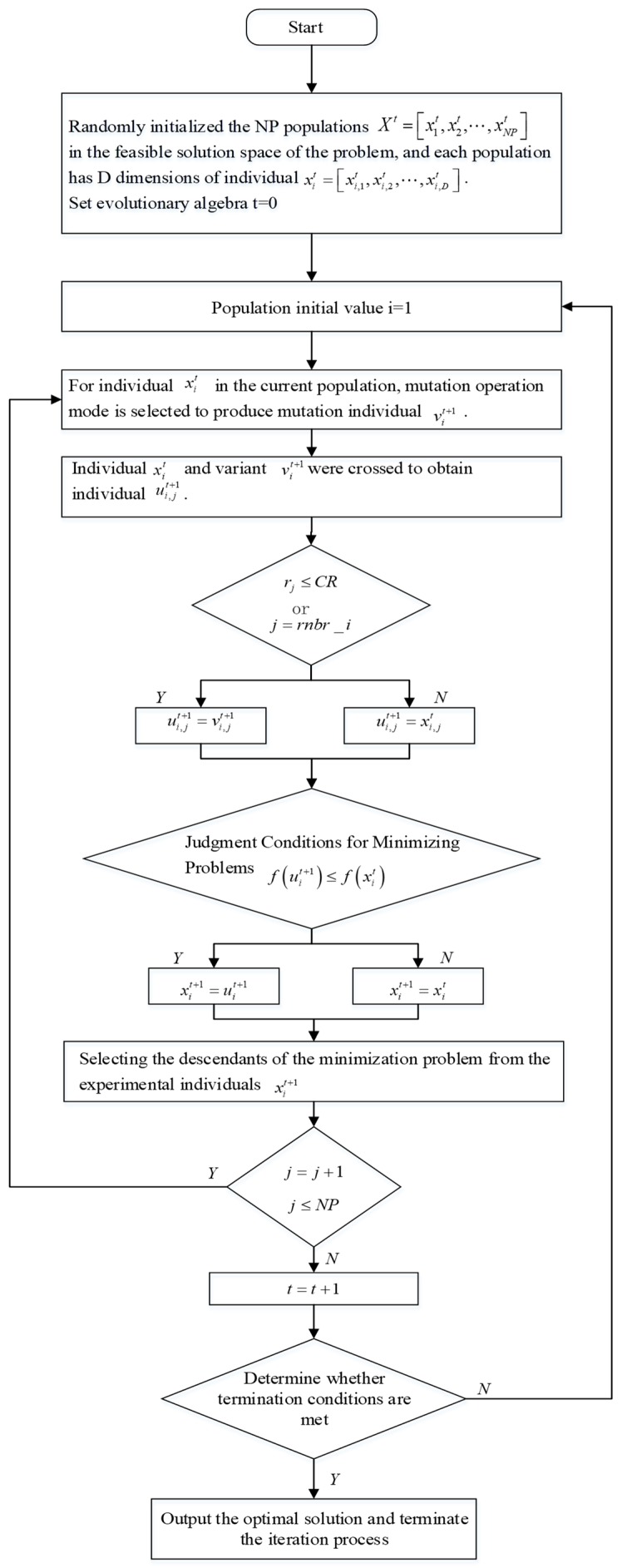
Flow chart of the differential evolution (DE) algorithm.

DE uses the greedy operation to select between $x_{i}^{t}$ and $u_{i}^{t+1}$, and generate the next generation of the individual $x_{i}^{t+1}$:

(12) $$x_{i}^{t+1}=\left\{ \begin{array}{ll} u_{i}^{t+1}\;, & if\;f\left(u_{i}^{t+1}\right)>f\left(x_{i}^{t}\right)\\ x_{i}^{t}, & otherwise \end{array}\right.$$

where f(⋅) is the objective function. Given that the optimization problem in this paper is in minimization form, the selection condition is $f\left(u_{i}^{t+1}\right)\leq f\left(x_{i}^{t}\right)$. The overall flow chart of the DE algorithm is shown in Figure 1.

## 4. Proposed DEIDV-Hop Algorithm

### 4.1. Goal

Given that the distance between the unknown node and the anchor node is an estimated value, the error is significant in the DV-Hop localization algorithm. In the original localization algorithm, it uses the unbiased estimation criterion to calculate the average distance per hop, and the estimation error of the average distance per hop achieved through this method is zero. However, the error complies with the Gaussian distribution. According to the parameter estimation theory, as the cost function of estimating the sub-error, it is more reasonable to use the mean square error than use only the variance or deviation. Therefore, the calculation formula of the average distance per hop is improved in this investigation.

### 4.2. Process of Flooding

Flooding, as a classic routing algorithm, is the simplest and most reliable one. Nevertheless, it excessively occupies network resources and generates a large amount of communication overhead, making routing and link resources inefficient.

The routing algorithms involved in the first and second step of the DV-Hop algorithm in this paper are based on the Flooding, including the original DV-Hop algorithm. To reduce network communication overhead, reduce network energy consumption as well as to extend the network life cycle, the EEDCF routing algorithm [42] is considered and applied.

### 4.3. Process of Calculating the Average Distance per Hop

From the traditional method on the unbiased estimation criterion to calculate the average distance of per-hop $HopSize_{i}$, let the value of the Equation (13) be zero.

(13) $$f_{1}=\frac{1}{m-1}\sum_{i\neq j}^{m}\left(d_{i,j}-HopSize_{i}\cdot h_{i,j}\right)$$

The minimum mean square error criterion is used to calculate the average distance per hop, and calculated by minimizing the Equation (14).

(14) $$f_{2}=\frac{1}{m-1}\sum_{i\neq j}^{m}{\left(d_{i,j}-HopSize_{i}^{\prime }\cdot h_{i,j}\right)}^{2}$$

Let $\frac{\partial f_{2}}{\partial HopSize_{i}^{\prime }}=0$, the estimated average distance per hop based on the minimum mean square error criterion can be obtained as:

(15) $$HopSize_{i}^{\prime }=\frac{\sum_{i\neq j}^{m}h_{i,j}d_{i,j}}{\sum_{i\neq j}^{m}h_{i,j}^{2}}$$

The average distance per hop is determined through Equation (16):

(16) $$HopSize{}^{\prime \prime }=\frac{\sum_{i=1}^{m}HopSize_{i}^{\prime }}{m}$$

After calculating $HopSize^{\prime \prime }$, each anchor node broadcasts its average distance per hop $HopSize^{\prime \prime }$ in the system through controlled flooding. When the unknown node u retrieves information from the anchor node i, it uses Equation (17) to determine the distance between the unknown node u and anchor node i.

(17) $$d_{u,i}^{\prime }=HopSize^{\prime \prime }\times h_{u,i}$$

### 4.4. Improvement of Differential Evolution Algorithm

In the basic differential evolution algorithm, three different individuals are randomly used to carry out mutation, crossover and individual update. This method is relatively simple, however, the update of individuals is unsighted without using the information of the optimal individuals obtained in the algorithm search that reduces the efficiency of the algorithm. Therefore, in the improved algorithm, the social part of PSO is introduced into the basic differential evolution algorithm. By learning from the optimal individual $X_{g}=\left(x_{g1},x_{g2},\cdots ,x_{gn}\right)$ in the population, the updated individual can obtain the optimal individual heuristic information and promote the convergence speed of the algorithm.

With the introduction of the heuristic information of the global optimal individual, individuals tend to converge to the global optimal individual with the operation of the algorithm, leading to the convergence of individuals and the decline of population diversity. To improve the global searching ability of the algorithm, the diversity of the population and the global searching ability are increased by improving the mutation and crossover operation, and they are presented next.

#### 4.4.1. Crossover

In the basic differential evolution algorithm, the target individual $x_{i}^{t}$ and the mutant individual $v_{i}^{t+1}$ are crossed to generate the experimental individual $u_{i}^{t+1}$. In the improved algorithm, the social learning part of the particle swarm optimization algorithm is introduced to make the current individual $x_{i}^{t}$ learn from the optimal individual $x_{best}^{t}$ of the group, and the results are crossed with the mutation vector $v_{i}^{t+1}$. By using the heuristic information carried by the group optimal value, the optimization efficiency of the algorithm is improved. The cross operation is as follows:

(18) $$u_{i,j}^{t+1}=\left\{ \begin{array}{l} v_{i,j}^{t+1},\;if\;r_{j}\leq CR\;or\;j=rnbr\_i\\ x_{i,j}^{t}+rand[0,1]\times c\times \left(x_{best}^{t}-x_{i,j}^{t}\right),\;otherwise \end{array}\right.$$

where c=2. $x_{i,j}^{t}+rand[0,1]\times c\times \left(x_{best}^{t}-x_{i,j}^{t}\right)$, and signifies that an individual $x_{i}^{t}$ learns from the optimal individual $x_{best}^{t}$ of the population.

#### 4.4.2. Selection

In the improved differential evolution algorithm, the diversity of the population and the global search ability are increased by carrying out small probability random variation on the variation vector. The operation of mutation is as follows:

(19) $$v_{i}^{t+1}=\left\{ \begin{array}{l} x_{j}^{\left(L\right)}+rand\left[0,1\right]\times \left(x_{j}^{\left(U\right)}-x_{j}^{\left(L\right)}\right)\;,\;if\;rand\left[0,1\right]\leq P_{r}\\ x_{r_{1}}^{t}+F\times \left(x_{r_{2}}^{t}-x_{r_{3}}^{t}\right),\;otherwise \end{array}\right.$$

$P_{r}$ is the probability of random variation. When the random number rand[0,1] is higher than the random mutation probability $P_{r}$, the mutation operation mode of the basic difference evolution algorithm is adopted; random mutation is carried out, if otherwise.

### 4.5. Differential Evolution Algorithm Implementation

In this paper, an optimized mathematical model is established by taking into consideration the distance between the unknown node and the anchor node, the location estimation process is simplified to minimize the optimization problem, and next, DE is used to provide a solution for the optimization model. First, the objective function is defined as:

(20) $$f\left(x_{u},y_{u}\right)=Min\sum_{i=1}^{m}\left|\sqrt{{\left(x_{u}-x_{i}\right)}^{2}+{\left(y_{u}-y_{i}\right)}^{2}}-d_{u,i}^{\prime }\right|$$

Among them, $\left(x_{u},y_{u}\right)$ is the coordinate of the unknown node, $\left(x_{i},y_{i}\right)$ is the coordinate of the anchor node, and $d_{u,i}^{\prime }$ is the estimated distance between the unknown node u and the anchor node i.

According to the aim function $f\left(x_{u},y_{u}\right)$, the fitness function is expressed as:

(21) $$\begin{array}{l} fitness\left(x_{u},y_{u}\right)\\ ={\sum_{u=1}^{n}\left(\frac{1}{h_{u,i}}\right)}^{2}f\left(x_{u},y_{u}\right)\\ \;={\sum_{u=1}^{n}\left(\frac{1}{h_{u,i}}\right)}^{2}\left(\sum_{i=1}^{m}\left|\sqrt{{\left(x_{u}-x_{i}\right)}^{2}+{\left(y_{u}-y_{i}\right)}^{2}}-d_{u,i}^{\prime }\right|\right) \end{array}$$

### 4.6. Complete DEIDV-Hop Algorithm

By combining Section 3 and Section 4, this paper proposes an enhanced DV-Hop algorithm based on DE called DEIDV-Hop, with the corresponding pseudo-code depicted in Algorithm 1.

The flow of the algorithm follows with DEIDV-Hop initializing network parameters and generating simulated network topology (random or grid topology) yet utilizing the shortest path method to retrieve the hop-count value between nodes, corresponding to lines 1–13. After that, the information on a hop-count value between nodes is retrieved, and the average distance per hop is calculated by Equation (16). Next, the estimated distance between unknown nodes and anchor nodes is calculated according to line 15. As the third step and corresponding to lines 16–36, the DE algorithm is utilized to estimate the location of unknown nodes by initializing the parameters of DE and have the maximum number of iterations set. It mutates the target individual selection Equation (18) in line 22, and Equation (19) is used for the cross operation to obtain test individuals in lines 23–25. The greedy criterion is used to select the next generation of individuals for the target and experimental individuals in lines 27–31; finally, the optimal individual in the group is the estimated location of the unknown node at the end of the iteration.
**Algorithm 1:** The procedure of DEIDV-Hop1: **Initialization:** total number of nodes N, Percentage p of anchor nodes, communication radius R;2: **Input:** Parameter settings of DEIDV-Hop and the experimental area;Parameter settings of DEIDV-Hop: see Table 1Experimental area is 100 × 100 m^2^3: Network deployment nodes to generate simulated network topology;4: Calculate the hop-count value $h_{i,j}$ according to the shortest path algorithm5: **for** k = 1 to N6:   **for** i = 1 to N7:    **for** j =1 to N8:     **if** short_path(i,k) + short_path(k,j) < short_path(i,j) 9:       short_path(i,j) = short_path(i,k) + short_path(k,j);10:      **end**11:    **end**12:   **end**13: **end**14: Calculate the average distance per hop, $HopSize^{\prime \prime }$ according to Equation (16);15: Calculate the unknown distances;16: **for** k = 1 to NP
17: **Initialization:** Generates NP individuals that contain 2 dimensions of variables according to Equation (9);18:   Calculate and evaluate each individual $x_{i}$;19:   t = 1;20:   **While**
$t<t_{max}$ do21:    **for**
i = 1 to NP //Mutation and Crossover22:     According to Equation (18), mutation vector $v_{i}^{t}$ is generated;23:     **for** j = 1 to D
24:      Trial vector $u_{i}^{t}$ was obtained by crossover according to Equation (19);25:     **end**26:     Trial vector $u_{i}^{t}$ was selected;27:     **if**
$f\left(u_{i}^{t}\right)<f\left(x_{i}^{t}\right)$ then //Minimize optimization problems28:      $x_{i}^{t+1}=u_{i}^{t}$;29:     **else**30:       $x_{i}^{t+1}=x_{i}^{t}$;31:     **end**32:   **end**33:    t=t+1;34:  **end**35:  The best individual is the location of the unknown node;36: **end**

## 5. Experimental Results and Analysis

**Figure 2:**
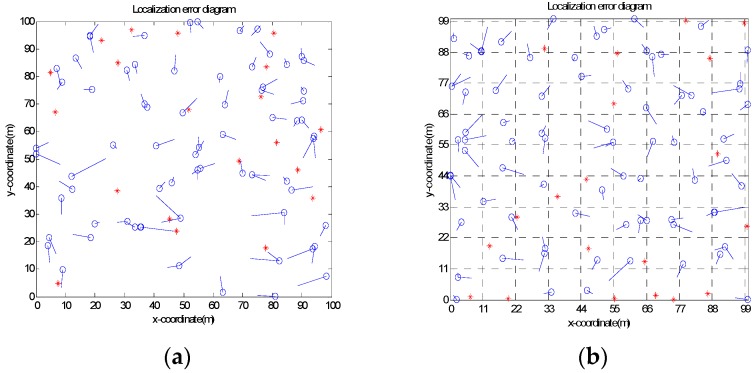

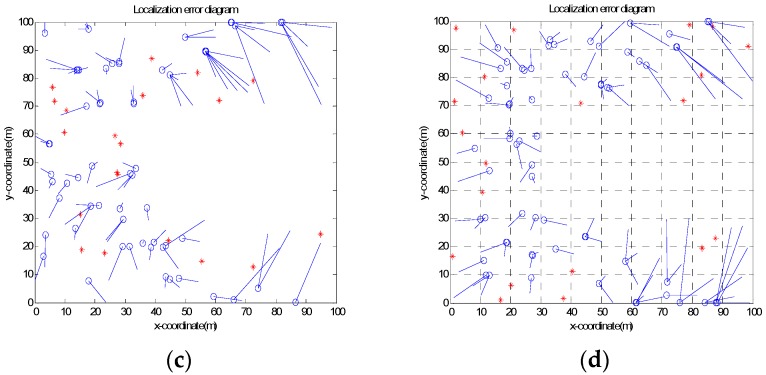
(**a**) Node error distribution diagram in square random topology; (**b**) Node error distribution diagram in a square grid topology; (**c**) Node error distribution diagram in C-Shaped random topology; (**d**) Node error distribution diagram in C-Shaped grid topology.

Experimental analyses are carried out under four different network simulation conditions to show the effectiveness of the proposed algorithm, listed as random topology, grid topology, C-Shaped random topology, and C-Shaped grid topology, as shown in Figure 2 respectively. In random topology, all nodes are randomly deployed in the corresponding experimental area, while in grid topology, each node is deployed at the intersection of gridlines and differentiated according to the proportion of the deployment deviation, in which ratio of 0 means that the nodes are precisely distributed at the gridline intersection. In comparison with random topology, the node distribution of grid topology is uniform.

When designing the C-shaped area, we considered excavating a part of the square area during the simulation. The cut-out area is 30 × 70 m^2^ (the original area was 100 × 100 m^2^).

The DEIDV-Hop algorithm is compared with DV-Hop, PSODV-Hop [43], and GSODV-Hop [44] through simulation implemented on MATLAB 2014a [45], running on a desktop PC with one Intel(R) Core(TM) i5-6500 CPU @3.20 GHz processor, 8 GB RAM, and Windows 7 OS.

In this investigation, only the two-dimensional coordinate plane is considered. All experimental results are averaged by running 100 times independently, and the size of the experimental area is 100 × 100 m^2^.

The localization error of unknown nodes u is:

(22) $$Error_{u}=\sqrt{{\left(x_{u}^{est}-x_{u}^{act}\right)}^{2}+{\left(y_{u}^{est}-y_{u}^{act}\right)}^{2}}$$

The average localization error (ALE) is taken as the evaluation criterion, and calculated as:

(23) $$Average\;Localization\;Error\left(ALE\right)=\frac{\sum_{i=1}^{n}\sqrt{{\left(x_{u}^{est}-x_{u}^{act}\right)}^{2}+{\left(y_{u}^{est}-y_{u}^{act}\right)}^{2}}}{n\times R}$$

where $\left(x_{u}^{est},y_{u}^{est}\right)$ and $\left(x_{u}^{act},y_{u}^{act}\right)$ is the estimated location and actual location of the unknown node u. ALE is the average localization error, N the total number of nodes, n is the number of unknown nodes.

In Equation (23), the numerator represents the Euclidean distance between the estimated location and the actual location of the unknown node, and the estimated error distance. This article includes R in the denominator, thus, Equation (23) with R is called the normalized average localization error.

Thus, n is given as:

(24) n=N−m

Experiments verify its influence on the average localization error in three aspects: the communication radius of nodes, the percentage of anchor nodes, and the total number of nodes. The parameters used in the DEIDV-Hop experiment are shown in Table 1, while the parameter settings for PSODV-Hop [43] and GSODV-Hop [44] are identical to original references and shown in Table 2 and Table 3, respectively.

The error graphs of nodes in four topologies, namely square random topology, square grid topology, C-Shaped random topology, and C-Shaped grid topology are listed in Figure 2. As settings for the investigation, the communication radius R of the node is 20 m, the total number of deployment nodes is 100, and the percentage of anchor nodes is 20%.

As shown in Figure 2, when the node deployment area changes from square topology to C-shaped topology, the average localization error of the node rises sharply. On the other hand, from the perspective of node distribution, random topology and grid topology can be considered a sparse network and intensive network. It can be observed that the nodes of the grid topology are evenly distributed if compared with the random topology; the error of nodes is smaller as well. As the node deployment changes from the random topology to the grid topology, the average localization error of the nodes decreases. The main reason is due to the influence of the deployment area, where the node distribution is not uniform and the hop-count value between nodes is no longer accurate.

### 5.1. Effect of Deployment Deviation Ratio λ in a Grid Topology

In this section, we study the effect of different deployment deviation factors on ALE for two grid topologies. When grid topology nodes are deployed, different values λ can be selected, λ∈[0,1]. As settings for the investigation environment, the total number of deployment nodes is 100, the percentage of anchor nodes is 20%, and the communication radius is 20 m. As discussed above, a smaller grid deployment deviation factor will have a better effect in the case of node deployment in a grid topology.

**Figure 3:**
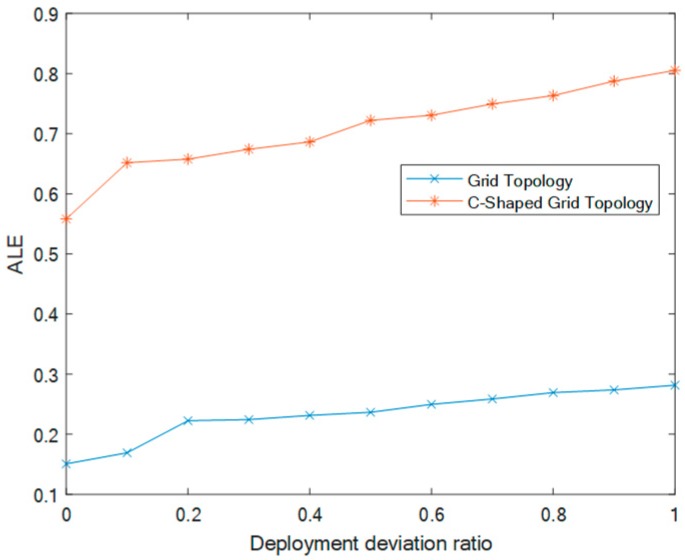
Effect of deployment deviation factors on two grid topology average localization error (ALE).

As shown in Figure 3, with the increase of λ, the ALE of DEIDV-Hop in two grid topologies also increases, though different λ values have a tiny influence on ALE results. When λ=0, the grid topology ALE is the smallest and the localization effect is the best. However, in practical production and living applications, it is challenging to deploy nodes with precision at fixed points, and often at random. In this investigation, λ=0.4 is adopted as a condition in the distribution of real network simulations, although other values such as 0.3 and 0.5 are also suitable.

### 5.2. Effect of Communication Radius on Average Localization Error (ALE)

**Figure 4:**
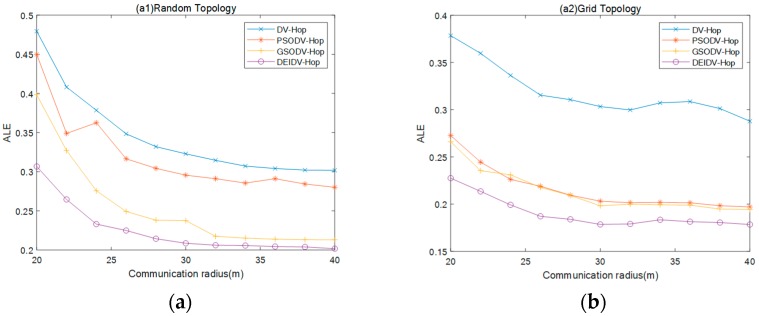

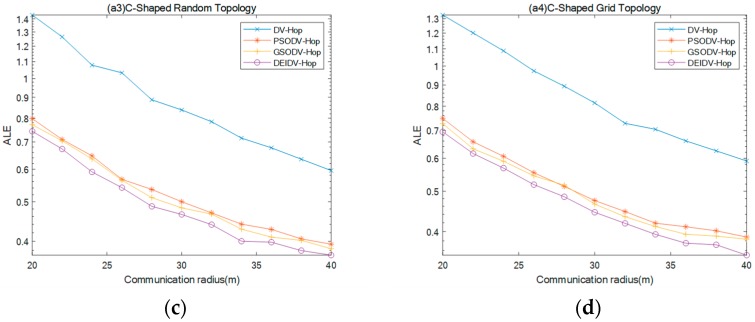
(**a**) Effect of communication radius on ALE in square random topology; (**b**) Effect of communication radius on ALE in a square grid topology; (**c**) Effect of communication radius on ALE in C-Shaped random topology; (**d**) Effect of communication radius on ALE in C-Shaped grid topology.

**Figure 5:**
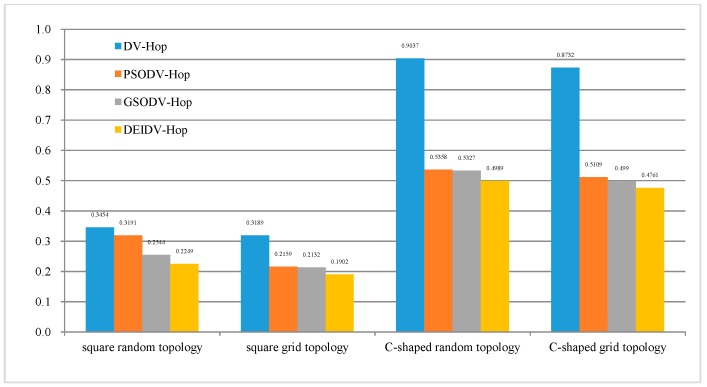
The average value of ALE for four topologies and algorithm.

In this section, as settings for the investigation, the communication radius R of the node varies from 20 m to 40 m, the total number of deployment nodes is 100, and the percentage of anchor nodes is 20%. Experimental results are shown in Figure 4 and Figure 5.

From Figure 4, it can be seen that, under the four network topologies, the average ALE on four localization algorithms gradually decreases with the increase of the communication radius of the nodes. Among these four algorithms, DEIDV-Hop always has the smallest ALE. As shown in Figure 5:

- PSODV-Hop reduces the average ALE by 7.61%, GSODV-Hop reduces by 26.35%, and DEIDV-Hop reduces by 34.88% when compared with DV-Hop in the square random topology,
- PSODV-Hop reduces the average ALE by 32.3%, GSODV-Hop reduces by 33.15% and DEIDV-Hop reduces by 40.36% when compared with DV-Hop in the square grid topology,
- Three meta-heuristic localization algorithms PSODV-Hop, GSODV-Hop, and DEIDV-Hop improve in average 40.71%, 41.05%, and 44.79%, respectively on localization accuracy when compared with DV-Hop in the C-Shaped random topology,
- Three meta-heuristic localization algorithms PSODV-Hop, GSODV-Hop, and DEIDV-Hop improve on average 41.49%, 42.85%, and 45.48%, respectively, on localization accuracy when compared with DV-Hop in C-Shaped grid topology.

With the increase of communication radius, the communication range of unknown nodes becomes significant; there will have more single-hop nodes that can establish direct contact with more adjacent nodes. Also, it reduces the hop-count value between some of the unknown nodes and anchor nodes. The average distance per hop estimated by the algorithm and the hop-count value between nodes also tends to be accurate. Therefore, the estimated distance between the unknown node and the anchor node is also more accurate, and a more accurate estimation of the location of the unknown nodes is obtained. The Euclidean distance between the estimated and actual location of the unknown node decreases, that is, the numerator of the ALE formula decreases, and the denominator R increases, which will inevitably bring about a decrease in the value of the entire formula.

In comparison with the random topology, the four algorithms under the grid topology have a significantly smaller average of ALE under the same conditions, which is caused by the denser distribution of network nodes. Due to the poor connectivity of nodes in sparse networks, some of the isolated nodes may be challenging to locate. The uneven distribution of nodes increases the hop-count value of unknown nodes from anchor nodes, and the estimated distance error also increases. The distribution of hop-count values between dense network nodes is relatively uniform, and the network connectivity between nodes is functional; thus, the average of ALE will be smaller.

### 5.3. Effect of the Percentage of Anchor Nodes on ALE

**Figure 6:**
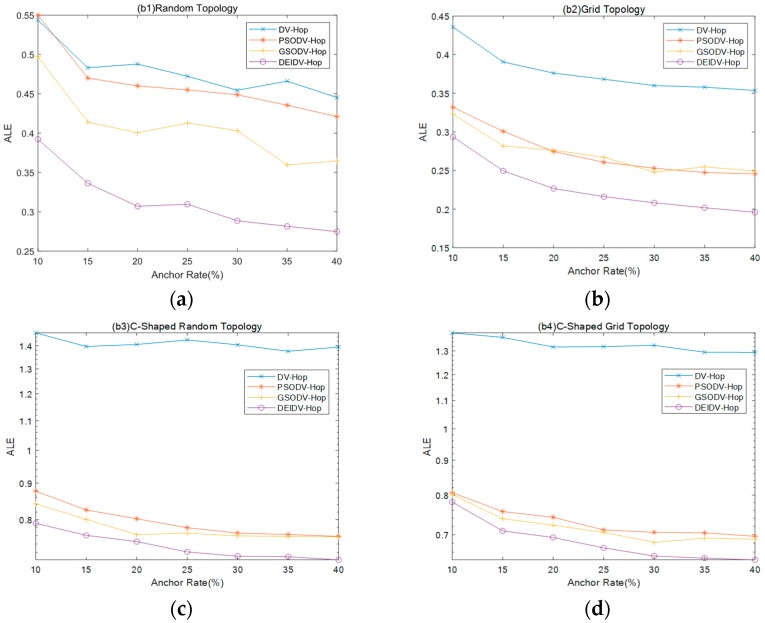
(**a**) Effect of the percentage of anchor nodes in square random topology on ALE; (**b**) Effect of the percentage of anchor nodes in square grid topology on ALE; (**c**) Effect of the percentage of anchor nodes in C-Shaped random topology on ALE; (**d**) Effect of the percentage of anchor nodes in C-Shaped grid topology on ALE.

**Figure 7:**
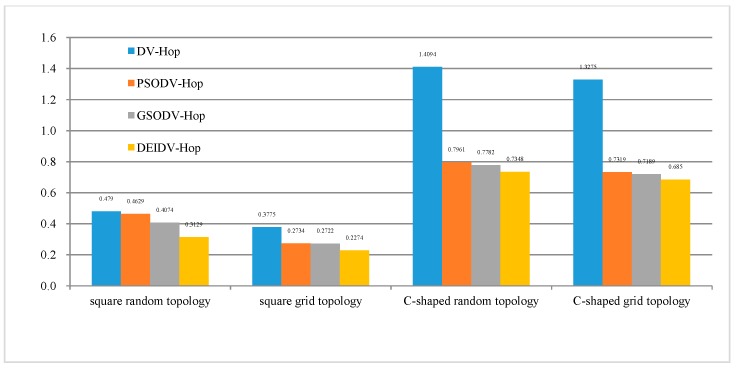
The average value of ALE for four topologies and algorithm.

In this section, as settings for the investigation, the percentage of anchor nodes gradually changes from 10% to 40%, with the number of deployment nodes 100 and communication radius of nodes 20 m. Experimental results are shown in Figure 6 and Figure 7.

As shown in Figure 6, the ALE of four algorithms decreases with the increasing anchor node ratio, and DEIDV-Hop always has the smallest ALE. As shown in Figure 7 and in comparison with DV-Hop:

- PSODV-Hop reduces the average of ALE by 3.36%, GSODV-Hop reduces by 14.95%, and DEIDV-Hop reduces by 34.68% in the square random topology,
- PSODV-Hop reduces the average of ALE by 27.58%, GSODV-Hop reduces by 27.89%, and DEIDV-Hop reduces by 39.76% in the square grid topology;
- Three meta-heuristic localization algorithms PSODV-Hop, GSODV-Hop, and DEIDV-Hop improve average 43.51%, 44.79%, and 47.86%, respectively, on localization accuracy in the C-shaped random topology;
- Three meta-heuristic localization algorithms PSODV-Hop, GSODV-Hop, and DEIDV-Hop improve average 44.87%, 45.85%, and 48.4%, respectively, on localization accuracy in the C-shaped grid topology.

With the increasing ratio of anchor nodes, there are more anchor nodes in the deployment area; accordingly the accuracy of the average distance per hop of anchor nodes is higher. DV-Hop is a calculation method based on the estimated distance, and the localization error decreases proportionally with the decrease of the estimated distance error. The increasing value of anchor nodes provides more favorable conditions for meta-heuristic localization algorithms and support to find the optimal solution. As a result, the average ALE is reduced because the location of nodes is accurately estimated.

### 5.4. Effect of Node Density on ALE

**Figure 8:**
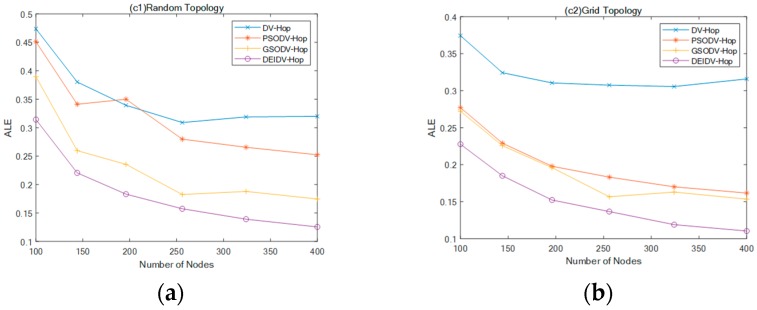

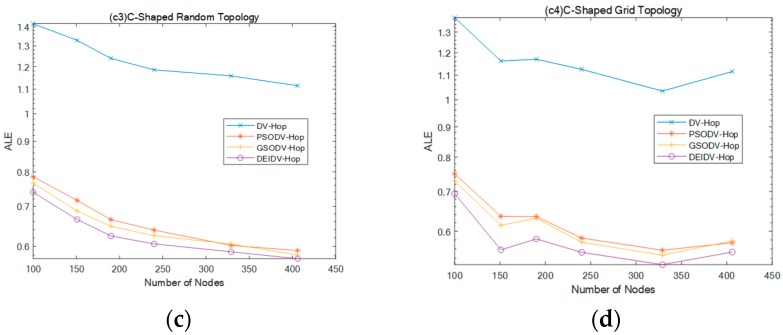
(**a**) Effect of the total number of nodes on ALE in square random topology; (**b**) Effect of the total number of nodes on ALE in a square grid topology; (**c**) Effect of the total number of nodes on ALE in C-Shaped random topology; (**d**) Effect of the total number of nodes on ALE in C-Shaped grid topology.

**Figure 9:**
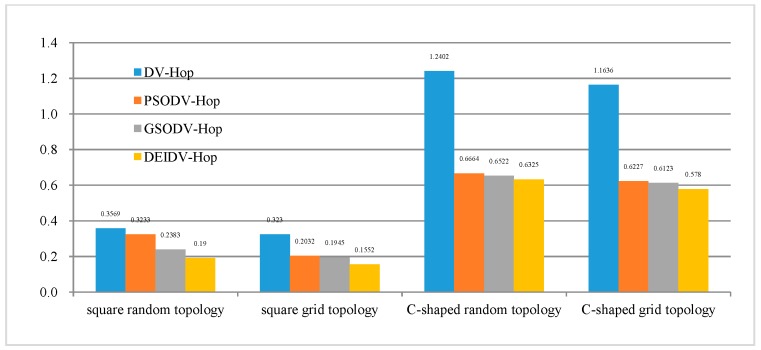
The average value of ALE for four topologies and algorithm.

In this section, the percentage of anchor nodes is 20% and the number of square topology deployment nodes is 100 to 400. Besides, the number of deployment nodes in the C-shaped deployment area is 100–406, and the communication radius 20 m. Experimental results are shown in Figure 8 and Figure 9.

As seen in Figure 8, as the percentage of anchor nodes increases, the ALE of the four algorithms decreases, and DEIDV-Hop always has the smallest ALE amongst the four network topologies. Using a comparison in Figure 9 with DV-Hop,

- PSODV-Hop reduces the average of ALE by 9.41%, GSODV-Hop reduces by 33.23%, and DEIDV-Hop reduces by 46.76% in the square random topology;
- PSODV-Hop reduces the average of ALE by 37.09%, GSODV-Hop reduces by 39.78%, and DEIDV-Hop reduces by 51.95% in the square grid topology;
- Three meta-heuristic localization algorithms PSODV-Hop, GSODV-Hop, and DEIDV-Hop improve average 46.25%, 47.41%, and 49%, respectively, on localization accuracy in the C-shaped random topology;
- Three meta-heuristic localization algorithms PSODV-Hop, GSODV-Hop, and DEIDV-Hop improve average 46.49%, 47.39%, and 50.33%, respectively, on localization accuracy in the C-shaped grid topology.

With the increase in the number of deployed nodes, the distribution of nodes becomes denser and tends to be in intensive networks. As each node has more single-hop connected nodes, the network connectivity is stronger. The deviation of the shortest path and straight line between nodes is relatively small, as is also the estimated distance error between the unknown nodes and anchor nodes. As an overall evaluation, with the increase in the number of deployed nodes, the average localization error of nodes also decreases, becoming more effective and productive.

## 6. Convergence Speed of the Algorithm and the Localization Error of Unknown Nodes

**Figure 10:**
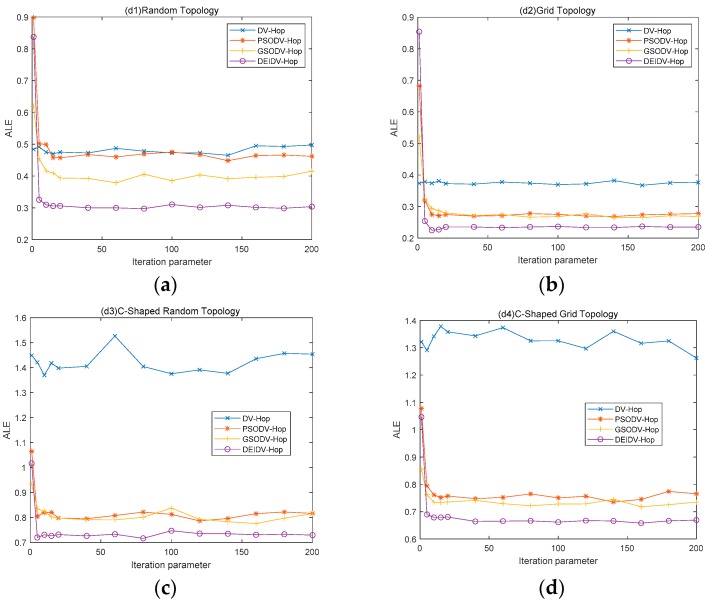
(**a**) Effect of the Iteration parameter on ALE in square random topology; (**b**) Effect of the Iteration parameter on ALE in a square grid topology; (**c**) Effect of the Iteration parameter on ALE in C-Shaped random topology; (**d**) Effect of the Iteration parameter on ALE in C-Shaped grid topology.

**Figure 11:**
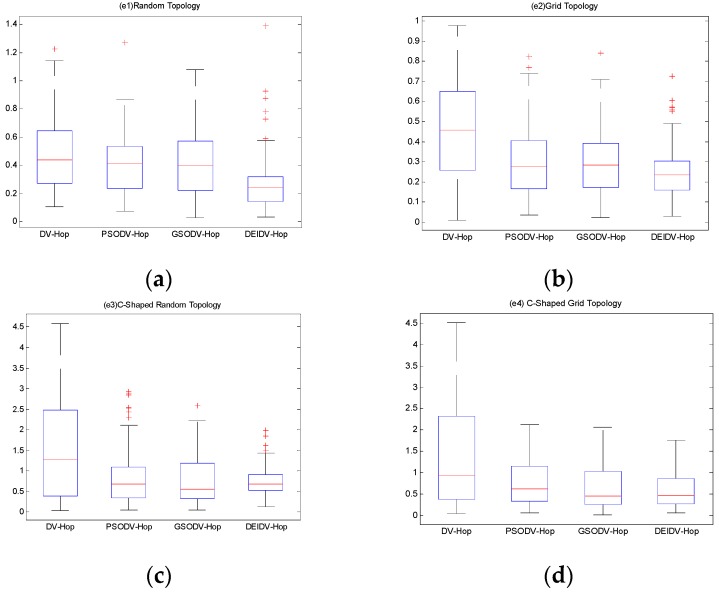
(**a**) The ALE of each unknown node in square random topology; (**b**) The ALE of each unknown node in a square grid topology; (**c**) The ALE of each unknown node in C-Shaped random topology; (**d**) The ALE of each unknown node in C-Shaped grid topology.

Experimental analysis was performed under four different network simulation conditions to prove the effectiveness of the convergence speed of the proposed algorithm and the localization error of each unknown node. The four network topologies are square random topology, square grid topology, C-shaped random topology, and C-shaped grid topology. Experimental results are shown in Figure 10 and Figure 11. The size of the experimental area is 100 × 100 m^2^.

### 6.1. Convergence Speed of the Algorithm

In this section, the percentage of anchor nodes is 20% and the number of deployment nodes is 100. The communication radius is 20 m. The number of iterations of the algorithm is 1–200. All experimental results are averaged by executing 100 times independently. Experimental results are shown in Figure 10.

According to the results of Figure 10, three meta-heuristic algorithms have faster convergence speeds yet better effects in the square topology. It can be clearly seen that the convergence speed of the three meta-heuristic algorithms is relatively fast; notably, the algorithm DEIDV-Hop proposed in this paper has a fast convergence speed and small localization error.

In the C-shaped topology, it is easy to see that the DV-Hop algorithm is very unstable. Because of changes in the deployment environment, there will be some isolated single-hop nodes, so the localization effect of the algorithm is very unstable. Looking at the curve in the Figure 10, the localization error values of the PSODV-Hop algorithm and GSODV-Hop algorithm continue to fluctuate with the increase of the number of iterations; in contrast, the DEIDV-Hop algorithm tends to have a stable value, which demonstrates the robustness of the algorithm.

The relationship between the number of iterations of the simulation algorithm and ALE in four topological environments proves that the DEIDV-Hop algorithm has fast convergence speed, robustness, and stability.

### 6.2. The Localization Error of Unknown Nodes

In this section, the percentage of anchor nodes is 20% and the number of nodes is 100. The communication radius is 20 m. Experimental results are shown in Figure 11. In this section, ALE≥1 is considered as an isolated node, and it is considered as a node that cannot be located.

As in Figure 11, boxplots are applied to show the following ALE results: minimum/maximum of the localization error, the median, and the 25th and 75th percentiles of the independent simulations of each unknown node in the algorithms of four network topologies. The DEIDV-Hop algorithm is relatively stable, and the localization error is lower than the original algorithms DV-Hop algorithm, PSODV-Hop algorithm, and GSODV-Hop algorithm,

- In the square random topology, there are four nodes in the DV-Hop algorithm that cannot be located, and 33 nodes are higher than the average of the localization error. In the PSODV-Hop algorithm, one node cannot be located, and the localization error of 44 nodes is higher than the average. In the GSODV-Hop algorithm, two nodes cannot be located, and the localization error of 38 nodes is higher than the average. In the DEIDV-Hop algorithm, one node cannot be located, and 28 nodes are higher than the average.
- In the square grid topology, among the four algorithms, no unknown node cannot be located. In the DV-Hop algorithm, the localization error of 41 nodes is higher than the average of the localization errors. The localization error of 36 unknown nodes in the PSODV-Hop algorithm is higher than the average. In the GSODV-Hop algorithm, the localization error of 36 nodes is higher than the average. In the DEIDV-Hop algorithm, the localization error of 36 nodes is higher than the average.
- In the C-shaped random topology, there are 46 nodes in the DV-Hop algorithm that cannot be located, and the localization error of 37 nodes is higher than the mean of the localization error. In the PSODV-Hop algorithm, there are 25 nodes cannot be located, and the localization error of 29 nodes is higher than the average. In the GSODV-Hop algorithm, 25 nodes cannot be located, and the localization error of 26 nodes is higher than the average. In the DEIDV-Hop algorithm, 14 nodes cannot be located, and the localization error of 36 nodes is higher than the average.
- In the C-shaped grid topology, there are 38 nodes in the DV-Hop algorithm that cannot be located, and the localization error of 34 nodes is higher than the mean of the localization error, where 26 nodes cannot be located and the localization error of 31 nodes is higher than the average in the PSODV-Hop algorithm. In the GSODV-Hop algorithm, 20 nodes cannot be located, and the localization error of 31 nodes is higher than the average. In the DEIDV-Hop algorithm, 18 nodes cannot be located, and the localization error of 30 nodes is higher than the average.

When the number of deployed nodes and the communication radius are the same, the four network topologies of the nodes will affect the distribution of the nodes. The grid topology will make the distribution of the nodes more uniform, denser, and tend to be intensive networks. In this way, each node has more single-hop connection nodes, and the hop-count value between nodes will become smaller and more regular. Thus, network connectivity is stronger. In random topology, the random distribution of nodes makes the hop-count value between nodes irregular, the connectivity between nodes deteriorates, and it tends to lead to sparse networks. There will be some isolated nodes that cannot be located, which increases the average localization error of nodes.

## 7. Conclusions and Future Work

In this paper, an enhanced algorithm for sensor node localization based on improved DV-Hop and DE Algorithms for WSNs is proposed, namely DEIDV-Hop. From evaluations and analysis, it is observed that the average distance per hop of anchor nodes is improved, confirming its advantages and efficacy.

In the localization estimation phase, the DE algorithm is introduced to locate unknown nodes, further reducing the localization error. To solve the problems of early maturity, low convergence, and low efficiency, the individual variation and cross operation formulas are improved. In the mutation operation, an attempt to increase the diversity of the population and the global search ability, the mutation vector is randomly mutated. Moreover, the social learning part of PSO is introduced in the crossover operation, and the optimization efficiency of PSO is improved by using the heuristic information carried by the optimal group value. Simulation results of the four network topology environments show that the average localization error of nodes can be effectively reduced by the DEIDV-Hop algorithm. Without the need for additional hardware, it shows excellent advantages and is effective. The simulation results show that the DEIDV-Hop algorithm has the fastest convergence speed and is the most stable. So far, a two-dimensional node location is the constraint to discussions in this investigation. The way of applying the algorithm to conduct node location in a more sophisticated space-three-dimensional node location is a direction to consider in terms of future research. In addition, based on the existing research, how to accurately apply the improved routing algorithm to the DV-Hop algorithm and make appropriate improvements to the routing algorithm to reduce the communication overhead of wireless threaded sensor networks and extend the life cycle of wireless sensor networks should be considered.

## Figures and Tables

**Table 1:** Parameter settings of DEIDV-Hop.

Parameter	Value
Deployment area size	100 m × 100 m
Percentage of the anchor node	10–40%
Communication radius R	20–40 m
NP	20
F	0.9
CR	0.2
$t_{max}$	20
$P_{r}$	0.02

**Table 2:** Parameter settings of PSODV-Hop.

Parameter	Value
$C_{1}$	2.05
$C_{2}$	2.05
No of particles	20
$V_{\max}$	10
No of iterations	20

**Table 3:** Parameter settings of GSODV-Hop.

Parameter	Value
ρ	0.4
γ	0.6
β	0.08
$I_{o}$	5
$n_{t}$	5
N	100
